# Supplementary material for: Robot-Assisted Magnetic Resonance Imaging-Targeted versus Systematic Prostate Biopsy; Systematic Review and Meta-Analysis
Source: Cancers (Basel). 2023 Feb 13;15(4):1181. doi: 10.3390/cancers15041181 (PMC9954527; doi:10.3390/cancers15041181)
Supplement: Supplementary file 1 [file cancers-15-01181-s001.zip › cancers-2167263-supplementary.pdf]

Supplementary Figures

Study or Subgroup	RA-TB Events	RA-TB Total	RA-SB Events	RA-SB Total	Weight	Odds Ratio M-H, Fixed, 95% CI	Year
Kaufmann et al., 2016	29	55	19	55	15.7%	2.11 [0.98, 4.55]	2016
Kroenig et al., 2016	26	52	31	52	27.1%	0.68 [0.31, 1.47]	2016
Lee et al., 2020	50	145	50	145	57.2%	1.00 [0.62, 1.62]	2020
**Total (95% CI)**		**252**		**252**	**100.0%**	**1.09 [0.76, 1.56]**	
Total events	105		100				

Heterogeneity: $Chi^2 = 4.42$, df = 2 (P = 0.11); $I^2 = 55\%$

Test for overall effect: Z = 0.46 (P = 0.65)

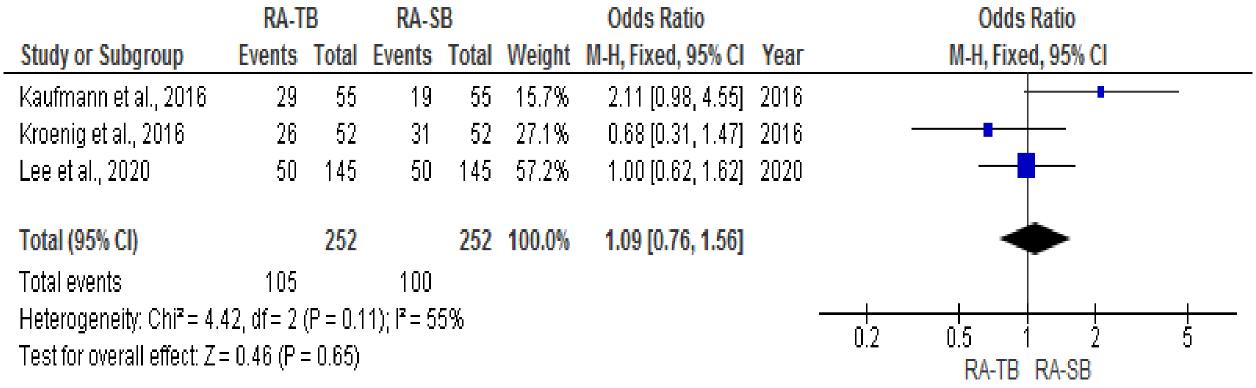

Figure S1. Cancer detection rate per prior negative patient

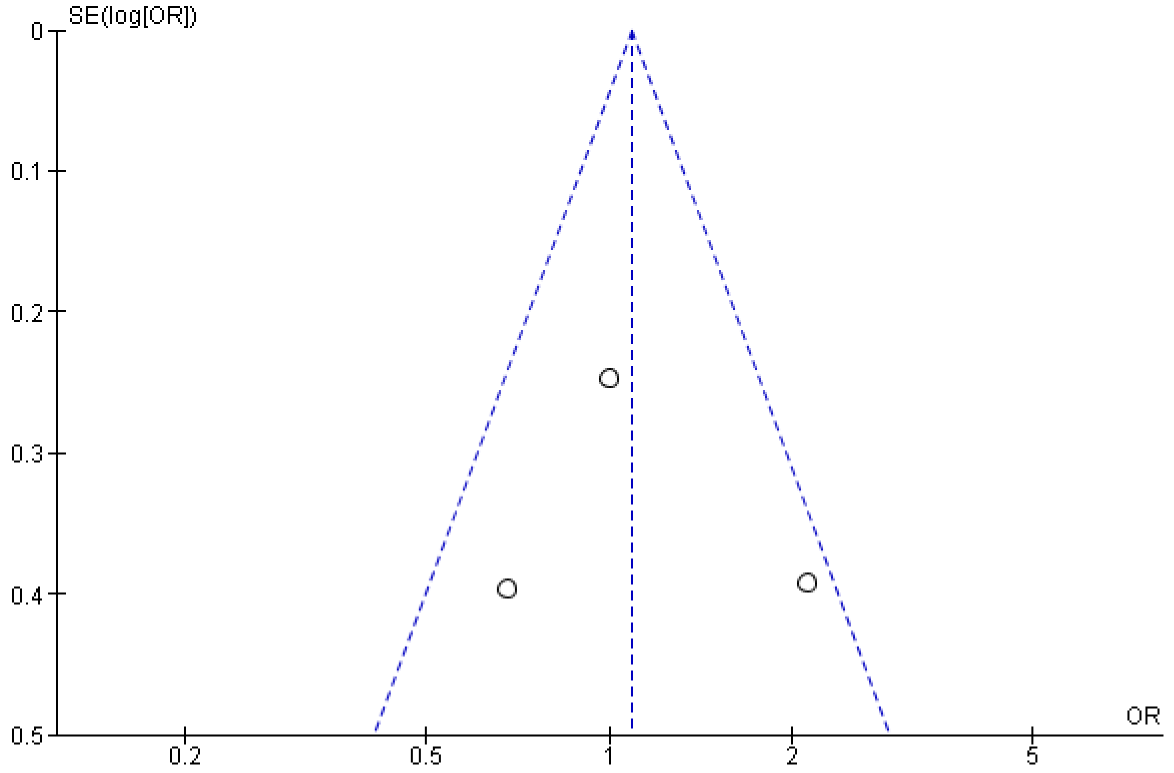

26

Figure S2. Funnel plot for cancer detection rate per prior negative patient

	RA-TB		RA-SB			Odds Ratio	
Study or Subgroup	Events	Total	Events	Total	Weight	M-H, Fixed, 95% CI	Year
Kaufmann et al., 2016	28	55	14	55	13.8%	3.04 [1.36, 6.79]	2016
Kroenig et al., 2016	23	52	26	52	29.0%	0.79 [0.37, 1.72]	2016
Lee et al., 2020	36	145	38	145	57.2%	0.93 [0.55, 1.58]	2020
**Total (95% CI)**		**252**		**252**	**100.0%**	**1.18 [0.81, 1.72]**	
Total events	87		78				
Heterogeneity: Chi² = 7.10, df = 2 (P = 0.03); I² = 72%							
Test for overall effect: Z = 0.86 (P = 0.39)							

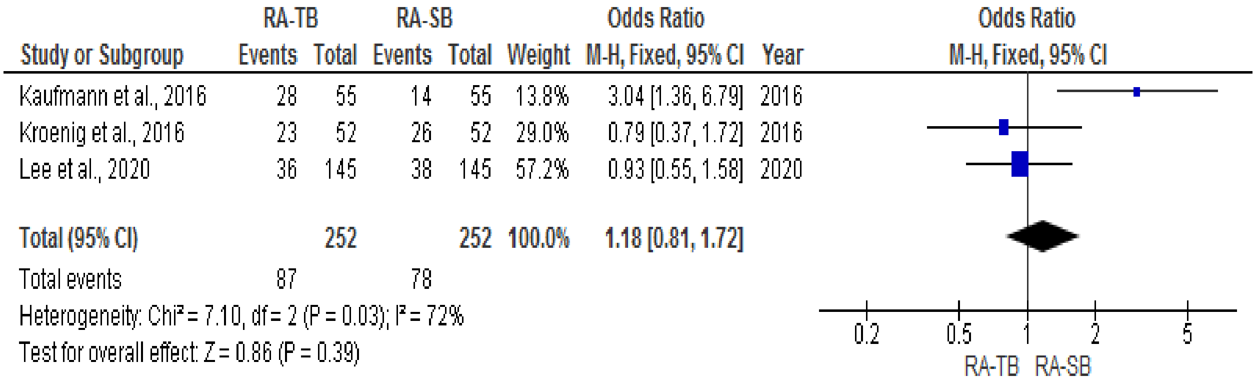

Figure S3. Clinically significant cancer detection rate per prior negative patient

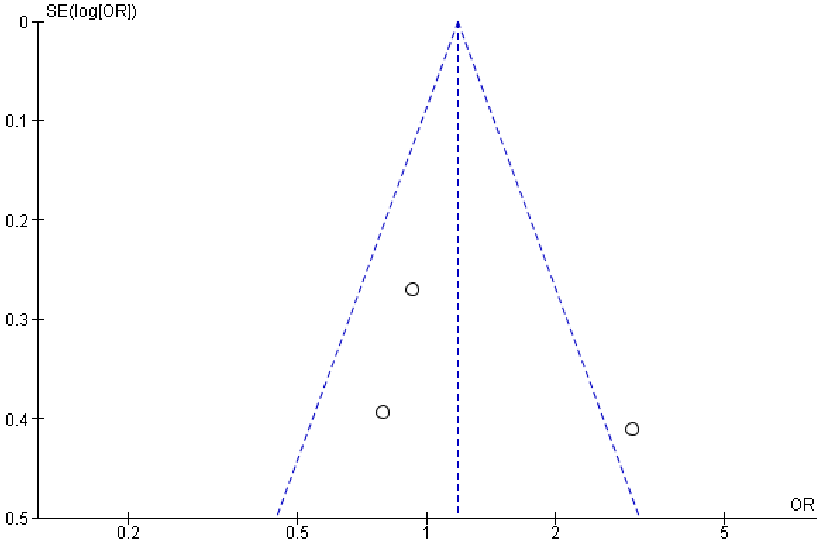

Figure S4. Funnel plot for clinically significant cancer detection rate per prior negative patient

	RA-TB		RA-SB			Odds Ratio	
Study or Subgroup	Events	Total	Events	Total	Weight	M-H, Fixed, 95% CI	Year
Kaufmann et al., 2016	1	55	5	55	15.8%	1.64 [0.61, 4.39]	2016
Kroenig et al., 2016	3	52	5	52	11.9%	0.58 [0.13, 2.54]	2016
Lee et al., 2020	12	145	8	145	72.3%	0.93 [0.55, 1.58]	2020
**Total (95% CI)**		**252**		**252**	**100.0%**	**1.00 [0.64, 1.55]**	
Total events	51		51				
Heterogeneity: Chi² = 1.57, df = 2 (P = 0.46); I² = 0%							
Test for overall effect: Z = 0.00 (P = 1.00)							

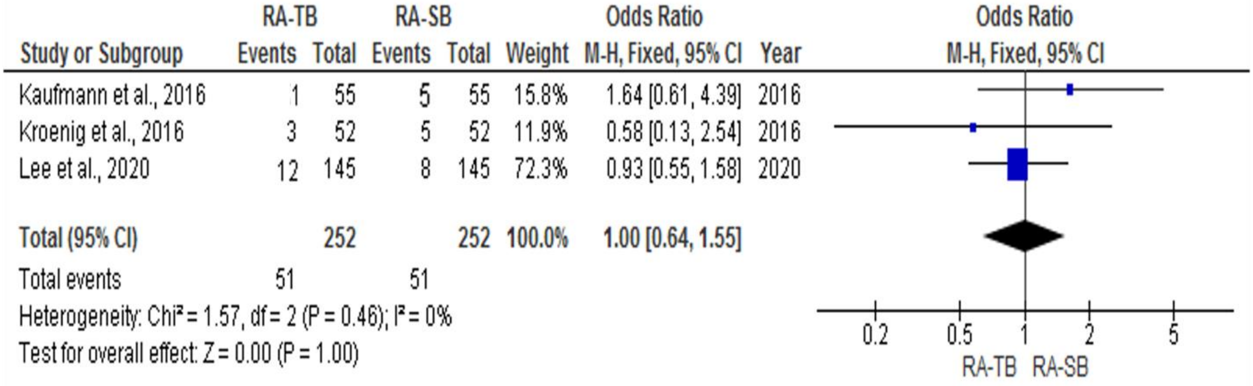

Figure S5. Clinically insignificant cancer detection rate per prior negative patient

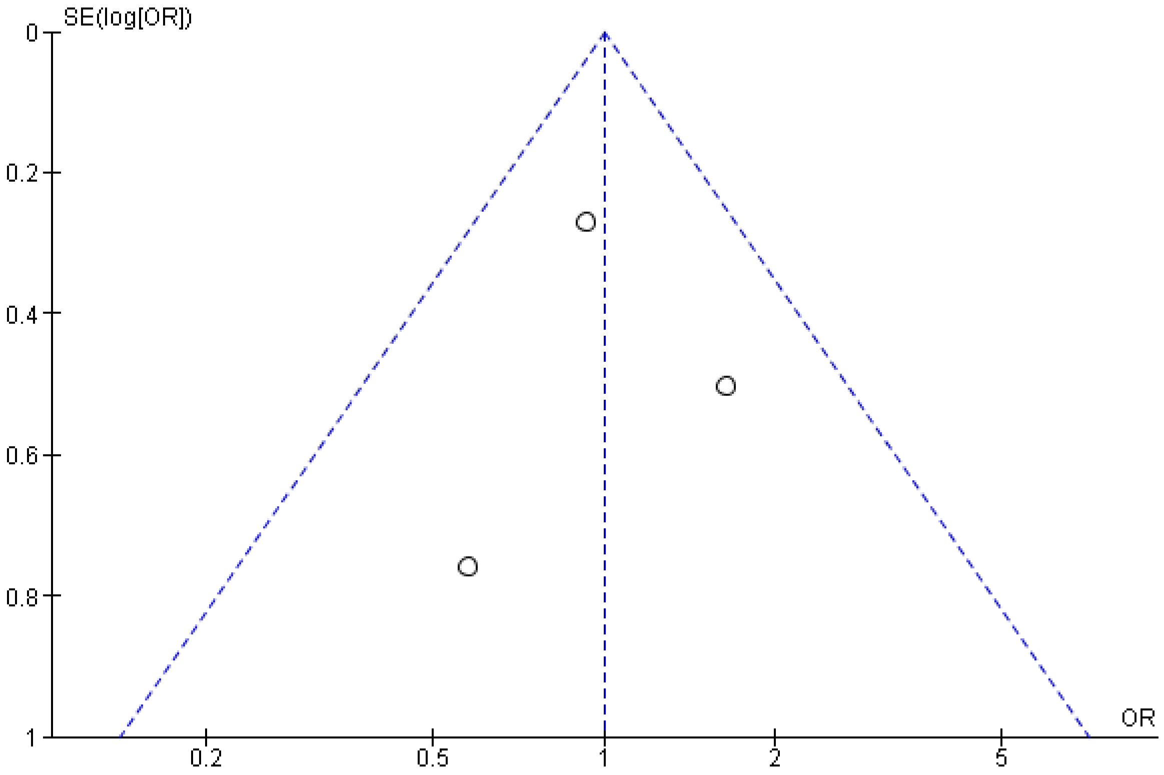

Figure S6. Funnel plot for clinically insignificant cancer detection rate per prior negative patient
